# The Nuclear Receptor—Co-repressor Complex in Control of Liver Metabolism and Disease

**DOI:** 10.3389/fendo.2019.00411

**Published:** 2019-06-26

**Authors:** Ning Liang, Tomas Jakobsson, Rongrong Fan, Eckardt Treuter

**Affiliations:** ^1^Department of Biosciences and Nutrition, Karolinska Institutet, Huddinge, Sweden; ^2^Department of Laboratory Medicine, Karolinska Institutet, Huddinge, Sweden

**Keywords:** hepatocytes, nuclear receptor, co-repressor, NCOR, HDAC3, GPS2, NAFLD, NASH

## Abstract

Hepatocytes are the major cell-type in the liver responsible for the coordination of metabolism in response to multiple signaling inputs. Coordination occurs primarily at the level of gene expression via transcriptional networks composed of transcription factors, in particular nuclear receptors (NRs), and associated co-regulators, including chromatin-modifying complexes. Disturbance of these networks by genetic, environmental or nutritional factors can lead to metabolic dysregulation and has been linked to the progression of non-alcoholic fatty liver disease (NAFLD) toward steatohepatitis and even liver cancer. Since there are currently no approved therapies, major efforts are dedicated to identify the critical factors that can be employed for drug development. Amongst the identified factors with clinical significance are currently lipid-sensing NRs including PPARs, LXRs, and FXR. However, major obstacles of NR-targeting are the undesired side effects associated with the genome-wide NR activities in multiple cell-types. Thus, of particular interest are co-regulators that determine NR activities, context-selectivity, and associated chromatin states. Current research on the role of co-regulators in hepatocytes is still premature due to the large number of candidates, the limited number of available mouse models, and the technical challenges in studying their chromatin occupancy. As a result, how NR-co-regulator networks in hepatocytes are coordinated by extracellular signals, and how NR-pathway selectivity is achieved, remains currently poorly understood. We will here review a notable exception, namely a fundamental transcriptional co-repressor complex that during the past decade has become the probably most-studied and best-understood physiological relevant co-regulator in hepatocytes. This multiprotein complex contains the core subunits HDAC3, NCOR, SMRT, TBL1, TBLR1, and GPS2 and is referred to as the “NR-co-repressor complex.” We will particularly discuss recent advances in characterizing hepatocyte-specific loss-of-function mouse models and in applying genome-wide sequencing approaches including ChIP-seq. Both have been instrumental to uncover the role of each of the subunits under physiological conditions and in disease models, but they also revealed insights into the NR target range and genomic mechanisms of action of the co-repressor complex. We will integrate a discussion of translational aspects about the role of the complex in NAFLD pathways and in particular about the hypothesis that patient-specific alterations of specific subunits may determine NAFLD susceptibility and the therapeutic outcomes of NR-directed treatments.

## Introduction

The liver is composed of multiple cell types, mainly hepatocytes and immune cells, and it is the major organ of glucose and lipid metabolism (1). Its metabolic potency and homeostasis is primarily coordinated at the level of gene expression via transcriptional networks composed of transcription factors (TFs, > 1,500) and co-regulators (>350). Disturbance of theses transcriptional networks by genetic, environmental, or nutritional factors can lead to dysregulated lipid and glucose metabolism and has been linked to the progression of non-alcoholic fatty liver disease (NAFLD) (2, 3). NAFLD is characterized by abnormal liver lipid accumulation and ranges from simple steatosis to non-alcoholic steatohepatitis (NASH), depending on the extent of inflammation and injury. The irreversible transition to liver fibrosis and cancer is the major cause of death in NASH patients, and there are currently no approved drugs for NASH therapy largely due to our insufficient understanding of the disease initiation and development (2, 3). Therefore, major efforts in the field are dedicated to identify key factors which promote or prevent the progression of NAFLD.

Amongst the key factors that have been discovered and validated in the past decades are many glucose- and lipid-sensing TFs. These include, but are not limited to, members of the nuclear receptor (NR) family such as oxysterol-sensing liver X receptors (LXRs), bile acid-sensing farnesoid X receptors (FXRs), and fatty acid-sensing peroxisome proliferator-activated receptors (PPARs), further TF sensors such as sterol regulatory element-binding protein 1c (SREBP1c) and carbohydrate-responsive element-binding protein (ChREBP) (2). In particular the PPARs and FXR have reached clinical significance with synthetic ligands being tested in phase III clinical trials (4, 5). However, major obstacles of TF-targeting drugs are the undesired side effects associated with the genome-wide role of TFs in positively and negatively regulating transcription in a highly context-dependent manner. Therefore, it is necessary to further dissect and better understand the mechanisms of gene-, cell type-, and signal-specific TF action to maintain the beneficial therapeutic outcomes of TF-targeting drugs while eliminating their side effects.

Of particular interest are TF-interacting co-regulators that determine the TF activities and the associated chromatin states at specific gene loci. Co-regulators have been generally classified into co-repressors and co-activators based on their effects on gene expression, but often they function highly context-specific either positively or negatively (6). Stimulating signals trigger the exchange of co-repressor and co-activator complexes at the TF-containing regulatory chromatin elements, mainly promoters and enhancers, to initiate the transcription process. The functional interaction of co-regulators with TFs and chromatin is highly gene- and cell-type-specific, and in some scenarios defines the altered sensitivity of cellular responses to extracellular stimuli linked with the progression of human diseases. In addition, as many co-regulators have a broad target range, their regulatory activities can be amplified through multiple TFs and other co-regulators, adding to their physiological impact. Further, most co-regulators form larger chromatin-modifying multiprotein complexes that are recruited to chromatin via TFs to modulate the epigenome, i.e., the DNA and histone modification status of chromatin.

**Figure 1:**
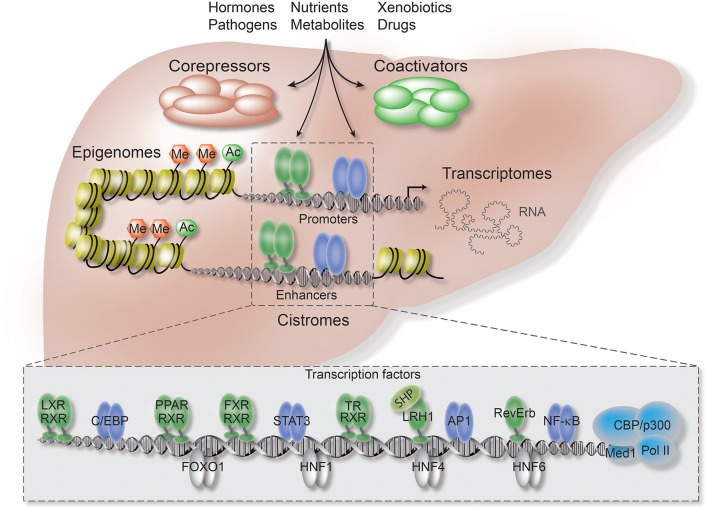
Co-repressors are integral components of regulatory transcriptional networks in hepatocytes. Highlighted are the key components of hepatocyte networks which directly or indirectly interact with co-repressors. Co-repressors are recruited to cis-regulatory chromatin elements, enhancers and promoters, by different classes of TFs, including metabolic NRs (*in green*), inflammatory TFs (*in purple*), and hepatocyte lineage-determining TFs (*in gray*). Both TFs and co-repressors respond to multiple activating and repressing extracellular signals and thereby transform signaling inputs into changes in gene expression and physiological pathways. Co-regulators function within multi-protein complexes that carry enzymatic activities entirely responsible for the sum of PTMs, including (de-)acetylation (Ac) and (de-)methylation (Me), of histones, TFs, and co-regulators. Hepatocyte-specific loss-of-function models, including conditional KO mice, have revealed first insights into the liver functions and target range of co-repressors, including the core subunits of the fundamental NR co-repressor complex. ChIP-seq has been applied to identify hepatocyte cistromes, i.e., the genome-wide binding sites of TFs and co-regulators, as well as epigenomes, i.e., the genome-wide chromatin modifications such as active and repressive histone marks. TFs and co-regulators play distinctive roles in linking cistrome and epigenome to determine the signal-regulated hepatocyte transcriptome, i.e., coding and non-coding RNAs, and thereby gene expression and physiological outcomes.

In contrast to the ongoing intense studies of TF function, current research on co-regulators is still limited by technical obstacles, including the lack of high-quality antibodies, combined with the fact that co-regulators unlike TFs do not bind DNA directly. Therefore, it remains poorly understood how the co-regulator/TF switch is coordinated by extracellular signals, such as hormones and inflammatory cytokines, and how the cell-, signal-, and gene-selective action of co-regulators is determined through TF-dependent and -independent mechanisms. In terms of liver physiology and disease, such understanding is extremely important since hepatocytes are constantly exposed to chemicals, nutrients and hormones (Figure 1).

**Figure 2:**
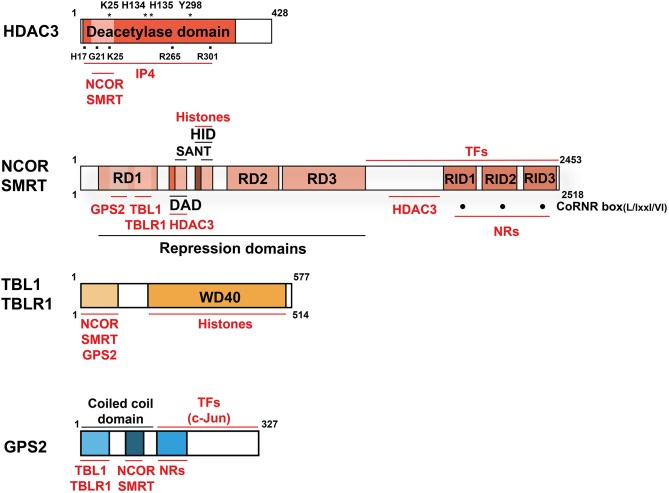
Domain structure and interactions of the co-repressor complex core subunits. Structural domains are labeled in BLACK and interacting proteins and molecules are labeled in RED. Indicated is the size (aa) of the human proteins (wild-type, full-length cDNAs of major isoforms). For HDAC3, mutations of several aa, marked with (*), abolish deacetylase activity. The aa marked with (■) are involved in the HDAC3-IP4 interaction. For NCOR and SMRT, three C-terminal CoRNR-boxes (·) mediate NR-interactions and thus serve as RIDs. Note that the numbering of the RIDs in NCOR and SMRT differs in individual publications and no consensus nomenclature exists. NCOR, SMRT, and GPS2 interact directly also with other TFs than NRs, but the interaction domains are poorly characterized. NCOR/SMRT and TBL/TBLR1 interact additionally with hypoacetylated histones, thereby stabilizing chromatin interactions of the entire complex. Key abbreviations: DAD, deacetylase-activating domain; HID, histone-interacting domain; RD, repression domain; RID, NR-interacting domain; IP4, inositol (1, 4–6) tetraphosphate.

**Figure 3:**
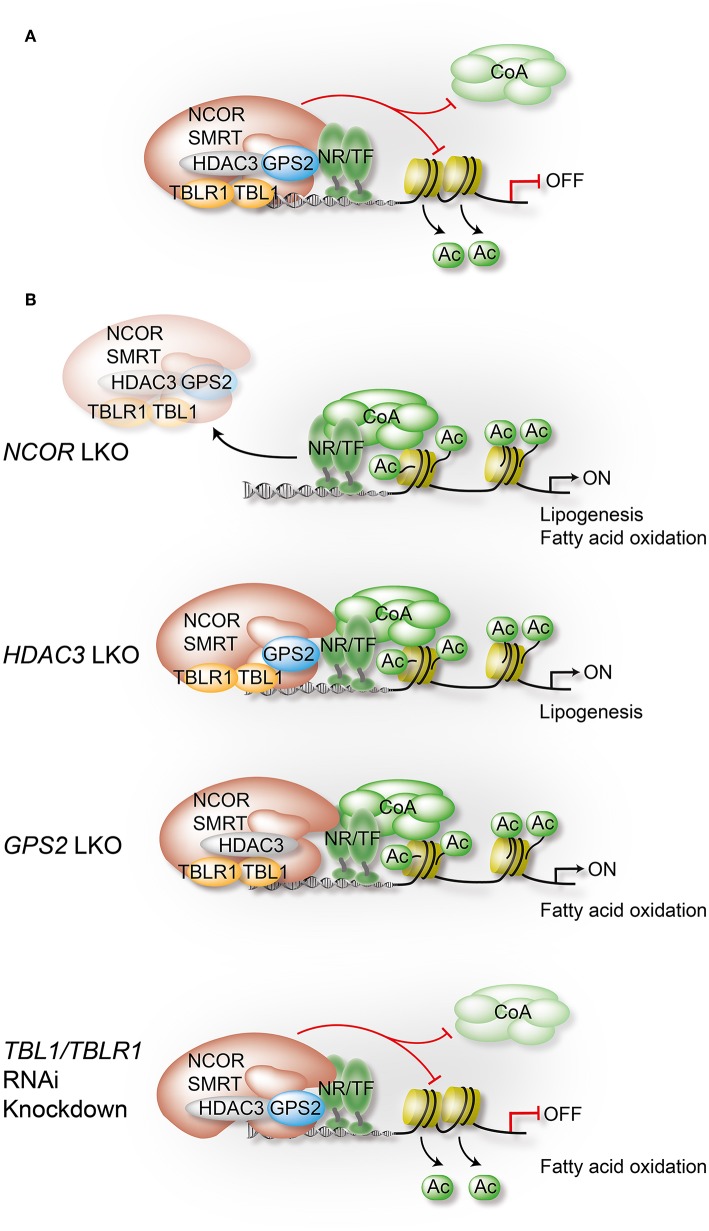
Liver-specific depletion of individual co-repressor complex subunits reveals overlapping yet non-redundant roles. In hepatocytes, NCOR, to a lesser extent SMRT, and GPS2 directly interact with TFs, including metabolite-sensing NRs and inflammatory TFs. TBL1/TBLR1 may stabilize chromatin recruitment of the complex via histone binding, and they are involved in PTM-regulated co-repressor-co-activator exchange mechanisms. Hepatocyte HDAC3 requires NCOR interactions to access TFs and chromatin, and its repressive function is in part deacetylase-independent, suggesting a regulatory role beyond histone deacetylation. Highlighted are in **(A)** the assembly of the core subunits of the co-repressor complex in WT hepatocytes, and in **(B)** the alterations that occur upon depletion of a specific subunit using conditional KO mice or RNAi knockdown in liver. Models are derived from studies that have usually focused on the characterization of one individual subunit, rather than characterizing the entire complex. Although a complex-independent function of a given subunit cannot be excluded, the comparison of the results from different studies strongly supports the involvement of the complex, and perhaps functional sub-complexes, in most of the identified pathways and loss-of-function phenotypes. All highlighted examples are discussed and cited in the text.

While many general and physiological aspects of co-regulator function have been reviewed recently (6–8), we will here focus on one particular co-repressor complex. During the past decade, individual subunits of this multi-protein complex have become the probably most-studied and best-understood physiological relevant co-regulators in the liver. Core subunits are histone deacetylase 3 (HDAC3), nuclear receptor co-repressor (NCOR, also known as NCOR1), and silencing mediator of retinoic acid and thyroid hormone receptor (SMRT, also known as NCOR2). Thus, the complex is usually referred to as the HDAC3 complex, the NCOR/SMRT complex, or simply as NR-co-repressor complex. Additional core subunits are G-protein pathway suppressor 2 (GPS2), transducin β-like protein 1 (TBL1, also known as TBL1X), and TBL-related 1 (TBLR1, also known as TBL1XR1) (8–15) (Figures 2, 3A).

There could be several reasons for the fundamental importance of this co-repressor complex for liver physiology and disease. A first reason could be that the majority of target TFs for the complex are NRs, which are the most abundant and crucial metabolic sensors and physiological regulators amongst the liver TFs. In fact, the TF-binding subunits of the complex (NCOR, SMRT, GPS2) have all been initially identified by means of two-hybrid protein-protein interaction screenings using liver NRs as bait, in part using liver cDNA libraries (16). A second reason could be that hepatocyte-specific knockout (KO) mice, mutations, and RNAi-mediated knockdown models, have been generated for most of the subunits. This enabled researchers to study the physiological consequences of loss-of-co-repressor-function *in vivo* under physiological conditions and in disease models, but it also revealed insights into the genomic action and TF target range of co-repressors. Table 1 summarizes the published mouse models that deplete or mutate individual subunits, and other relevant co-repressors, in hepatocytes. A third reason could be related to the particular feasibility of mouse liver hepatocytes, as compared to other metabolic tissues such as pancreatic islets or distinct adipose tissue depots, for the preparation of high-quality chromatin suitable for next-generation sequencing-based approaches such as chromatin immunoprecipitation-coupled sequencing (ChIP-seq) (56).

**Table 1:** Summary of loss-of-function mouse models revealing liver-specific corepressor functions and target TFs.

**Protein name (gene name)**	**Mouse model**	**Key features of the phenotype**	**Target TF**	**References**
NCOR (*Ncor1*)	Global KO	• Embryonic lethality at E15.5 • Impaired erythroid, thymocyte, and CNS development	–	(17)
	Liver NCORi transgenic	• Overexpression of a dominant-negative NCOR blocked basal transcription of TR-responsive genes but had no effect on ligand-activation • Increased endogenous SMRT and NCOR mRNA expression • Increased hepatocyte proliferation in euthyroid mice	TR	(18)
	Liver NCOR ΔRID knockin	• Increased expression of TR positive targets in both hypothyroid and euthyroid conditions • Improved cholesterol tolerance due to diminished intestinal cholesterol absorption (as the result of changes in the composition and hydrophobicity of the bile salt pool)	TR	(19–21)
	Global NCOR ΔRID knockin	• Increased energy expenditure as a result of enhanced sensitivity to TH • Could rescue insulin-resistant phenotype of mutant TRβ	TR	(22, 23)
	Global DADm knockin	• Leaner due to increased energy expenditure • Improved insulin-sensitivity • Abnormal circadian behavior due to aberrant regulation of clock genes • Altered oscillatory patterns of several metabolic genes • Derepressed TH-activated genes in euthyroid and hypothyroid mice liver	TR LXR	(24, 25)
	LKO (AAV8-TBG-Alb-Cre)	• Developed hepatosteatosis due to increased lipogenesis	RevErb LXR	(26)
	LKO (Alb-Cre)	• Repressed lipid synthesis in the fasting state • Repressed fatty acid oxidation and ketogenesis in the feeding state • Improved liver regeneration after partial hepatectomy and blocked diethylnitrosamine (DEN)-induced hepatocarcinogenesis	PPARα LXR ERRα	(27, 28)
	LKO (Alb-Cre)	• Developed hepatosteatosis due to increased lipogenesis	TR	(29)
SMRT (*Ncor2*)	Global KO	• Embryonic lethality before E16.5 due to lethal heart defect • Impaired neural development in forebrain • Fail to maintenance of the neural stem cell state	RAR	(30)
	Global SMRT mRID knockin	• Decreased energy expenditure • Developed global glucose tolerance and insulin sensitivity • Increased adiposity due to enhanced adipogenesis • Impaired type I pneumocytes differentiation and produced respiratoty distress syndrome at birth	TR PPARγ	(31)
	Global SMRTmRID1 knockin	• Accelerated aging (reduced mitochondrial function and increased susceptibility to oxidative stress) • Developed global glucose intolerance and insulin resistance • Upon HFD: • Obesity, insulin-insensitive, and refractory to the glucose lowering effects of TZD and AICAR, energy metabolism shifts from OxPhos to glycolysis • Mesenteric adipose tissue: adipocyte hypertrophy and increased inflammatory • Liver: hepatosteatosis • BAT: reduced thermogenic capacity and mitochondrial biogenesis	RAR TR PPARα PPARγ	(32, 33)
	LKO (AAV8-TBG-Alb-Cre)	• No obvious metabolic phenotype	–	(26)
	LKO (Alb-Cre)	• Little effect on most of TR targets in either euthyroid or hypothyroid animals • de-repressed RAR targets (*Cyp26a1*)	RAR	(29)
NCOR/SMRT (*Ncor1/2*)	NCOR/SMRT LKO (Alb-Cre)	• Hepatosteatosis due to activated hepatic lipogenesis and lipid storage • Normal glucose sensitivity • Increased ChREBP isoforms expression	TR RAR	(29)
	Global NS-DADm knockin	• Upregulated lipid-metabolic genes and mile hepatosteatosis • Undetectable HDAC3 enzyme activity, abrogated genome-wide HDAC3 recruitment, as well as increased local histone acetylation level	Lipid-sensing NRs	(34)
HDAC3 (*Hdac3*)	Global KO	• Embryonic lethality before E9.5		(35)
	LKO (Mx1-Cre plus pIpC injection or Alb-Cre)	• Hepatomegaly due to hepatocyte hypertrophy • Hepatosteatosis • Increased serum TG, total serum cholesterol, and LDL • Hypersensitive to insulin	PPARγ2	(36)
	LKO (AAV8-TBG-Alb-Cre)	• Alteration in circadian genes • Hepatosteatosis due to increased lipogenesis and sequestration • Repressed gluconeogenesis • Improved insulin sensitivity	RevErb HNF4α HNF6	(26, 34, 37–41)
TBL1 (*Tbl1x*)TBLR1 (*Tbl1xr1*)	Liver RNAi knockdown (adenovirus- delivered shRNA)	• Hepatosteatosis • Highly elevated VLDL TG • Inhibited of PPARα activity under both normal and HFD conditions	PPARα	(42)
GPS2 (*Gps2*)	Global KO	• Embryonic lethality around E10		(43)
	LKO (Alb-Cre)	• Dramatically reduced VLDL TG • Protected from HFD-induced hepatic steatosis and MCD-induced fibrosis • Enhanced PPARα-induced fatty acid oxidation	PPARα	(44)
PROX1 (*Prox1*)	Liver RNAi knockdown (AAV8-TBG-shRNA)	• Significantly elevated hepatic TG	HNF4	(41)
	LKO (Alb-Cre)	• Hepatic injury • Non-obese but insulin-resistant • Suppressed glycolysis • Upregulated both oxidative phosphorylation and autophagy	Lipid-sensing NRs	(45)
RIP140 (*Nrip1*)	Global KO	• Lean and resistance to HFD-induced obesity and hepatic steatosis • Increased oxygen consumption • Unaffected adipogenesis, increased certain lipogenic enzymes, and UCP1 in Adipose tissue • Inhibited lipogenesis and enhanced gluconeogenesis	LXR	(46, 47)
	Liver RNAi knockdown (adenovirus- delivered shRNA)	• Alleviated hepatic steatosis in tumor-bearing, cachectic animals • Increased free fatty acid oxidation and ketogenesis • Enhanced VLDL secretion • Reduced peripheral lipoprotein lipase activity	LXR PPARα	(48)
SHP (*Nr0b2*)	Global KO	• Increased bile acids synthesis under chow diet • Dietary bile acids induce liver damage and restore feedback regulation • A synthetic FXR agonist is not hepatotoxic and has no regulatory effects • Cholestyramine enhanced the expression of *CYP7A1* and *CYP8B1* and reduced the bile acid pool	LRH1 HNF4α LXR	(49)
	Global KO	• Increased bile acids synthesis under chow diet • No significant defects in cholesterol metabolism under chow diet • Bile acids still can suppress *Cyp7a1* expression • Resistant to bile acid induced liver damage	LRH1 HNF4α LXR	(50, 51)
	Liver-specific overexpression	• Depleted of hepatic bile acid pool • Accumulated TG in liver	FXR LRH1 HNF4α PPARγ SREBP1c	(52)
	LKO (Alb-Cre)	• De-repressed *Cyp7a1* and *Cyp8b1* under chow diet and cholesterol and cholic acid diet • Resistance to diet induced hypercholesterolemia	Multiple NRs	(53)
	FXR SHP DLKO	• Cholestasis and liver injury as early as 3 weeks of age due to dysregulation of bile acid homeostatic genes • Activated C21 steroid biosynthesis pathway • Lower hepatic TG accumulation, improved glucose/insulin tolerance, and accelerated fatty acid use in aged mice	Multiple NRs	(54, 55)

## NCOR But Not SMRT Is Critical for Repression in Hepatocytes

### Identification, Structure, and Repression Mechanisms Support a Crucial Role in NR-Pathways

NCOR (57, 58) and SMRT (59–61) were first identified based on their interaction with unliganded NRs, including thyroid hormone receptors (TR), retinoic acid receptors (RAR) and the orphan receptor RevErb. Both proteins are extremely large (molecular weight around 270 kDa), which is suitable for forming a scaffold-binding surface for simultaneous interactions with target TFs, co-regulators and histone modifiers to form a co-repressor complex. NCOR and SMRT have a significant sequence homology (43%) and share an overall structure containing conserved functional domains (62, 63). The NCOR/SMRT N-terminus contains several independent repression domains (RD) (57, 64). GPS2 and TBL1/TBLR1 interact with distinct conserved regions of the RD1 to form a three-way core complex (11–13). In addition, TBL1/TBLR1 associate with RD3 via WD-40 repeats (12). Two SANT (SW13/ADA2/NCOR/TFIIB)-like domains locate between RD1 and RD2. HDAC3 directly binds to the deacetylase-activating domain (DAD), composed of a DAD-specific motif and one SANT domain (11, 65). The histone-interacting domain (HID), containing the other SANT domain, preferentially recognizes hypoacetylated histone tails and synergizes with the DAD to promote histone deacetylation and target gene repression (66). DAD binding is critical for recruitment and activation of HDAC3 and also for subsequent TR repression (34, 65, 67). At the C-terminal regions of NCOR and SMRT, three separate receptor-interacting domains (RIDs) have been identified to interact with the ligand-binding domains (LBD) of unliganded or antagonist-bound NRs (58, 68). The RIDs consist of conserved CoRNR peptide motifs that have the consensus sequence L/IxxI/VI (L: leucine, I: isoleucine, V: Valine, X: any aa) (69) (Figure 2). Since the RIDs differ in their binding affinities to individual NRs *in vitro*, they may determine the overall NR-affinity of NCOR and SMRT *in vivo* (32). While the global KO of NCOR or SMRT in mice caused embryonic lethality (17, 30), a variety of transgenic overexpression, knockin, and KO mouse models, carrying different mutations of NCOR and SMRT, have been instrumental to dissect the *in vivo* function and target range of these corepressors.

### A Truncated NCOR Lacking Repression Domains Acts Dominant Negative

A truncated human NCOR variant lacking its repressing domains had been initially cloned using two-hybrid screenings with TRβ (70). When expressed in the CV-1 cell line, this truncated NCOR abolished repression on positive TH-response elements. A likely interpretation is that NCOR lacking the N-terminal RDs (NCORi) still binds to TR due to the intact RIDs but fails to repress, thus acting dominant negative to interfere with repression by endogenous NCOR and SMRT (70). This was further confirmed *in vivo* using hepatocyte-specific expression of a dominant-negative NCORi in several transgenic mouse lines (18). NCoRi selectively de-repressed the basal expression of TR-target genes, while TH-dependent transcriptional activation of these genes was unaffected. Interestingly, proliferation of hepatocytes was increased in these mice, indicating that NCOR may also modulate transcription of other signaling pathways including those linked to cellular proliferation.

### NCOR/SMRT RID Mutant Knockin Mice Reveal NR-Selective Repression Pathways

Hepatocyte-specific NCORΔRID knockin mice were generated to express a mutant NCOR lacking the RIDs, thus a NR-binding deficient NCOR. Analysis of these mice revealed that repression of TR was abrogated both in the absence and presence of ligand. This indicated that an intact NCOR is required to repress unliganded TR and determines the magnitude of the TR ligand response (19). It also suggested that SMRT cannot fully compensate for the loss of NCOR binding to TR in hepatocytes. Besides the liver, the global abrogation of TR-NCOR interaction increased the thyroid hormone (TH) sensitivity in multiple tissues, with higher expression of TR targets in the presence of identical or lower levels of circulating TH (22). Crossing of these mice with TRβ mutant mice, a model of TH resistance in which TRβ is unable to release NCOR properly, rescued the TH resistance phenotype, demonstrating the critical role of NCOR in central and peripheral TH action *in vivo* (23). NCOR may regulate multiple NR signaling pathways in the liver since LXR target genes were also enhanced in NCORΔRID mice (19, 20). Upon feeding with a high-cholesterol diet, NCORΔRID mice had improved dietary cholesterol tolerance by altering bile acid metabolism to decrease bile pool hydrophobicity and diminish intestinal cholesterol absorption. However, some of these beneficial effects on hepatic cholesterol clearance were independent of LXRα since it was observed in both WT and LXRα KO mice. It was suggested that changes could be due to the TRβ1-induced *Cyp27a1* and *Cyp3a11*, two enzymes in the bile acid synthesis pathway, and canalicular bile salt pump *ABCB11*, indicating the NCOR-TR interaction in regulation of bile acid synthesis and hepatic cholesterol clearance (20).

Global mutant RID knockin (mRID) mice have been also generated for SMRT (31). These mice exhibited widespread metabolic defects including reduced respiration, systemic glucose intolerance, and insulin resistance. Adiposity was severely increased in these mice, consistent with PPARγ de-repression which would increase adipogenic capacity and accelerate white adipose tissue differentiation. In addition, similar to the NCORΔRID phenotype, some TR target genes in the livers were de-repressed (31).

Other mouse models were generated to investigate the NR-binding preferences of individual SMRT RIDs *in vivo*. To study the function of RID2, suggested to bind PPARs, mRID1 knockin mice were generated (32). On one hand, this model may reflect a gain-of-function since the SMRT interactions and repression of RID2-associated NRs, such as PPARs, will be reinforced. Indeed, PPAR target genes involved in fatty acid catabolism and oxidative phosphorylation were repressed by SMRT mRID1, resulting in reduced mitochondrial function and increased susceptibility to oxidative stress (32, 71). Notably, SMRT mRID1 mice developed premature aging, hyperlipidemia and insulin resistance, underscoring the role of SMRT in modulating metabolic rates and aging-related metabolic diseases (32). On the other hand, SMRT mRID1 mice may also be seen as a loss-of-function model because the RID1 mutation impairs SMRT interactions with TR and RAR. Although apparently normal when fed with a chow diet, upon a high-fat diet (HFD) mRID1 mice became super-obese with a striking lipid accumulation predominantly in adipose tissue depots. The mice developed multiple metabolic dysfunctions, as evidenced by adiposity-associated inflammation as well as systemic insulin resistance. SMRT mRID1 mice were also resistant to the glucose lowering effects of insulin-sensitizing drugs such as TZDs and AICAR, indicating that the RID1 is needed to protect the mice from diet-induced insulin resistance. In the livers of SMRT mRID1 mice, HFD increased the expression of PPARγ and LXRα but decreased SHP, resulting in induction of lipogenic genes and *Cyp7a1*. Thus, hepatic and serum triglycerides (TG) and cholesterol levels were dramatically increased. All these observations suggest that RAR, TR, and PPAR signaling pathways in the liver are impaired in NR-binding deficient SMRT mice.

### Single and Double KO Mice for NCOR and SMRT Reveal Non-redundant Roles

A number of liver-focused studies suggest a dominant physiological role of NCOR, along with HDAC3, in hepatocytes. Liver-specific depletion (LKO) of NCOR phenocopied the metabolic changes observed in HDAC3-depleted livers, including accumulation of hepatic lipids, reciprocal reduction of hepatic glycogen content, and up-regulation of hepatic lipogenesis (36, 37). Gene expression (transcriptome) profiling of NCOR LKO and HDAC3 LKO livers revealed similarity, since the upregulated genes were highly enriched in lipid and fatty acid metabolism, consistent with the lipid metabolic phenotypes (Figure 3B). Genome-wide chromatin occupancy of NCOR (i.e., the NCOR cistrome as determined by ChIP-seq) revealed a robust circadian rhythm in phase with HDAC3, whereas the hepatic SMRT cistrome did not oscillate. This suggested that NCOR may be more important than SMRT in recruiting HDAC3 to chromatin in hepatocytes (26, 38). Consistent with this, these studies show that liver-specific SMRT depletion did not cause obvious metabolic alterations. Therefore, extrahepatic tissues such as adipose tissue could be responsible for the metabolic alterations observed in heterozygous SMRT KO mice or knockin mice bearing mutations in the RIDs (31–34, 38). Interestingly, the hepatosteatosis phenotype observed upon hepatocyte-specific NCOR depletion in NCOR LKO mice is in contrast to the normal hepatic lipid content despite modest increased lipogenesis in mutant NCOR RID or DAD knockin mouse models (18, 19, 22–24, 34). These findings suggest that both interactions with NRs and enzymatic HDAC3 activation contribute to, but are not absolutely required for, NCOR function *in vivo*.

To determine the specific roles of NCOR and SMRT in liver TH signaling, mice have been generated that depleted or mutated NCOR, SMRT, or both in the liver. Surprisingly, deletion of liver SMRT under either euthyroid or hypothyroid conditions had little effect on TH signaling. In contrast, NCORΔRID mice confirmed the unique and sufficient role of NCOR in mediating TH sensitivity on positively regulated target genes. In addition, while SMRT LKO mice failed to activate pathways involved lipid synthesis and storage, SMRT loss strikingly potentiated NCORΔRID upregulated target genes including ChREBP (29). Interestingly, there was no evidence of glucose intolerance despite the increased hepatic steatosis when both corepressors were disrupted (29). This closely resembled the phenotype of hepatocyte-specific HDAC3 LKO mice (36, 37). Although there was no effect on TH signaling, liver SMRT loss specifically increased expression of certain RAR target genes, which were not changed in the NCORΔRID livers. Together, these data indicate that NCOR and SMRT display TF target selectivity *in vivo* and that NCOR may be the dominant NR co-repressor in hepatocytes. However, there are data suggesting that both co-repressors also cooperate to control lipogenic gene expression and hepatic lipid storage through the recruitment of HDAC3 and the regulation of specific NRs including TR (27).

### NCOR and mTOR Signaling

The mammalian target of rapamycin (mTOR)—signaling pathway plays a central role in regulating lipid metabolism in the liver (72). The liver-specific deletion of TSC1, an inhibitor of mTOR complex 1 (mTORC1), resulted in the constitutive activation of mTOR signaling, which in turn led to a pronounced defect in fast-induced ketone body production. In addition, livers from aged mice also developed defective ketogenesis accompanied by the increased mTORC1 activation, which can be corrected by depletion of hepatic RAPTOR, an essential mTORC1 component. This indicates a key role of mTOR activation in age- and fasting-related hepatic ketogenesis, a process which is also controlled by PPARα. How mTOR signaling influences PPARα activation has remained enigmatic until NCOR has been identified as mediator. It has been found that mTOR activation promotes the nuclear localization of NCOR, which then acts as the predominant co-repressor of PPARα in hepatocytes (73). Specifically, this effect has been attributed to S6 kinase 2 (S6K2), a downstream effector of mTORC1, which interacts with NCOR and controls its subcellular localization (73, 74). In line with this, S6K2 activity was elevated in *ob/ob* mice, a common genetic mouse model of obesity. Thus, this pathway identified a mechanism for how energy availability may direct influence the nuclear localization and action of a key co-repressor, thereby PPARα repression and hepatic ketogenesis (74).

### Signal-Regulated Phosphorylation of NCOR Modulates NR Pathway Selectivity

**Figure 4:**
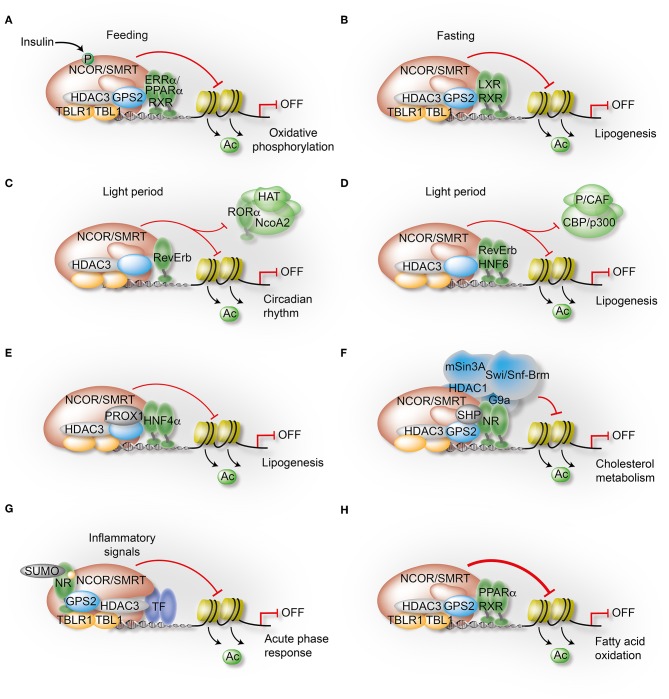
Mechanisms governing the transcriptional control of liver metabolism by variable co-repressor sub-complexes. **(A,B)** NCOR selects its targets depending on the cellular energy status. Insulin induces phosphorylation of NCOR serine 1460, which selectively favors NCOR interaction with PPARα and ERRα over LXRα. As a result, PPARα/ERRα-dependent oxidative metabolism is attenuated due to repression **(A)**, while LXRα-dependent lipogenesis is increased due to de-repression **(B)**. **(C)** RevErbα recruits the NCOR-HDAC3 complex to its canonical DNA-binding motifs to repress circadian clock genes during the light period. **(D)** Simultaneously, HNF6 recruits RevErbα and the associated co-repressor complex to mediate circadian repression of lipogenic gene expression during the light period. **(E)** Immuno-precipitation-coupled mass spectrometry from mouse liver extracts identified interactions of the HDAC3 complex with the homeodomain co-repressor PROX1, which mediates the recruitment to HNF4α-bound enhancers to repress lipogenesis. **(F)** The atypical orphan receptor SHP inhibits cholesterol metabolism by acting as a co-repressor for multiple NRs via distinct mechanisms, one involving interactions with the GPS2 subunit of the co-repressor complex. **(G)** GPS2 mediates trans-repression of the hepatic acute phase response by docking to SUMOylated LXRβ and LRH1. As a consequence, inflammatory signals fail to release the co-repressor complex from chromatin and inflammatory gene expression remains repressed. **(H)** GPS2 and NCOR synergistically interact with PPARα, thereby potentiating the repressive function of the co-repressor complex to inhibit PPARα-dependent fatty acid oxidation.

Strikingly, liver-specific NCOR depletion revealed apparently paradoxical phenotypes, indicating that NCOR may select its TF targets in a context-dependent manner according to the cellular energy status to orchestrate liver energy metabolism. On one hand, NCOR LKO mice suffered from hepatic steatosis as a consequence of enhanced hepatic lipogenesis and lipid storage, suggesting NCOR to repress lipogenic genes (18–20, 26). On the other hand, NCOR seemed critical for repressing PPARα-induced hepatic fatty acid oxidation and ketogenesis (73, 74). Interestingly, it was demonstrated that the insulin-Akt signaling pathway differentially modulates NCOR activity at genes linked to lipogenesis and ketogenesis/oxidative phosphorylation (OxPhos) during the feeding-fasting transition. Insulin induces NCOR phosphorylation at serine 1460, which selectively favors NCOR interaction with PPARα and ERRα over LXRα. As a result, NCOR phosphorylation causes de-repression of LXRα target genes to increase lipogenesis while, at the same time, still represses PPARα and ERRα target genes to attenuate oxidative metabolism in the liver (Figures 4A,B). This phosphorylation-dependent modulation of NCOR affinities to LXRα, PPARα, and ERRα may explain the apparent paradox that liver-specific deletion of NCOR concurrently induces both lipogenesis and oxidative metabolism. More generalized, post-translational modifications (PTMs) such as phosphorylation could provide an important mechanism by which co-repressors can switch targets and selectively modulate liver metabolism (27). Indeed, multiple phosphorylation sites, some of which are regulated by insulin-signaling, have been identified in NCOR and other core subunits in the context of mouse liver steatosis and insulin signaling (75).

## HDAC3 Controls Circadian Rhythm and Physiology Dependent and Independent of Its Enzymatic Activity

### Place of HDAC3 Within the Mammalian HDAC Superfamily

The mammalian HDAC superfamily comprises 18 members with a highly conserved deacetylase domain, which can be further classified into 4 classes based on their catalytic mechanism and sequence homology. Using inhibitor as an affinity tag, HDAC1 was first characterized as an ortholog of yeast Rpd3 (reduced potassium dependency-3) (76). Rpd3/Hda1 related zinc-dependent deacetylases are grouped into classes I, II and IV, whereas Sirtuin-related NAD^+^-dependent deacetylases constitute class III. Class I HDACs comprise HDAC1, HDAC2, HDAC3, and HDAC8, consist of the conserved deacetylase domain with short amino- and carboxy-terminal extensions, and are rather ubiquitously expressed (77). Class I HDACs form multiple-protein nuclear complexes that are recruited to chromatin through interaction with TFs and co-regulators (63). HDAC 1 and 2 are assembled into NuRD, Sin3, CoREST, and MiDAC complexes (77). Biochemical purification and mass spectrometry suggests that HDAC3 is mainly found in the NCOR/SMRT co-repressor complex. It is the enzyme responsible for histone deacetylation at regulatory promoters and enhancers, and it requires interactions with NCOR/SMRT to become enzymatically active (15) (Figure 2). HDAC3 should thus be particularly important for connecting the transcriptional and epigenetic functions of the NR corepressor complex in the liver.

### Mouse Models Reveal How HDAC3 Controls the Liver Clock, Physiology, and NAFLD

Hepatocyte-specific HDAC3 depletion using Alb-Cre (Cre expression under the control of albumin promoter, LKO mice) or AAV8-TBG-Cre (adeno-associated virus (AAV) serotype 8 expressing Cre under control of TBG promoter) revealed the pivotal role of HDAC3 in the regulation of the circadian rhythm as well as of hepatic lipid, cholesterol and carbohydrate metabolism in mice (36–38). HDAC3 depletion in liver significantly increased *de novo* lipogenesis and cholesterol synthesis, but decreased fatty acid oxidation, thus causing dramatically elevated hepatic and serum triglyceride and cholesterol levels, resulting in severe hepatosteatosis (36, 38) (Figure 3B). However, HDAC3 LKO mice concurrently showed improved glucose tolerance and insulin sensitivity. That is probably because intermediary metabolites were rerouted from hepatic gluconeogenesis to *de novo* lipogenesis, which in turn prevented lipotoxicity and hepatic insulin resistance (37).

A critical aspect of HDAC3 function was uncovered with the demonstration that its deacetylase activity depends on interaction with the DAD of NCOR or SMRT (26, 34). The HDAC3:SMRT-DAD crystal structure revealed an inositol (1, 4–6) tetraphosphate (IP4) sandwiched between the interface of HDAC3 and DAD to stabilize the complex (78). Consistently, mutations of residues that make critical IP4 contacts by forming hydrogen bonds and salt bridges (78), such as SMRT Y470A (corresponding to NCOR Y478A) and HDAC3 K25A (Figure 2), abolished the DAD-HDAC3 interaction and HDAC3 enzymatic activity *in vitro* and *in vivo*, as evidenced in the corresponding mutant mice (26, 34). Due to the abrogated HDAC3-DAD interaction by NCOR Y478A and SMRT Y470A mutations (NCOR/SMRT-DADm), genome-wide HDAC3 chromatin occupancy as well as histone deacetylation were significantly reduced in NCOR/SMRT-DADm mice. This suggested that the majority of HDAC3 is recruited to the genome via NCOR and SMRT (34). Although HDAC3 protein levels were constant over the 24 h light-dark cycle, genome-wide ChIP-seq analysis revealed that HDAC3 as well as NCOR displayed a rhythmic occupancy at regulatory regions of lipid synthesis genes, enriched in the light period (day) and diminished at the dark period (night). As HDAC3 occupancy was inversely correlated to H3K9 acetylation and RNA polymerase II recruitment at these gene loci, HDAC3 likely regulates lipogenesis by directing the rhythm of the epigenome, i.e., epigenetic modifications of chromatin. Consistent with this mechanism, the circadian rhythm was lost in HDAC3 LKO mice, causing elevated fatty acid synthesis and hepatic steatosis (38). Although not significantly increasing catabolic gene expression, the NCOR-DADm (Y478A) livers displayed phase shifts in the expression of several metabolic genes, increased energy expenditure and improved insulin sensitivity. This further confirmed the crucial role of HDAC3, and likely its enzymatic activity, in the epigenetic regulation of circadian and metabolic physiology (24).

### RevErb Is the Main Target for HDAC3 to Regulate the Liver Clock

In search for the involved target TFs, two core components of the circadian clock, the orphan receptors RevErbα and RevErbβ, were found to oscillate in phase with the chromatin recruitment of NCOR and HDAC3, thus likely the co-repressor complex. Importantly, the diurnal rhythm of recruitment was diminished in RevErbαβ-depleted livers, demonstrating that RevErb fully accounts for the circadian rhythmicity of co-repressor complex occupancy. The data imply that during the day, RevErb either directly binds to the RevDR2 and RORE DNA motifs at circadian clock genes, or it is tethered to DNA via HNF6 (38, 39) (Figures 4C,D). In both scenarios, the RevErb-recruited co-repressor complex is responsible for target gene repression, which involves maintaining local histone hypo-acetylation at regulatory loci. In contrast, during the night, when mice are active and feeding, the complex is released from chromatin due to the lowered concentrations of RevErb, allowing for co-activator recruitment and subsequent expression of clock and metabolic genes (39). In agreement with this mechanism, both RevErbα- and NCOR-deficient mice exhibit hepatosteatosis, resembling the HDAC3 LKO phenotype (26, 38). However, the HDAC3 LKO mice had much more severe hepatosteatosis than those lacking the two RevErbs, strongly suggesting that the HDAC3 co-repressor complex interacts with additional TFs, including NRs, to control the expression of genes involved in lipid homeostasis independently of RevErbs (40).

### HDAC3 Alterations During Aging Trigger Steatosis in Mice

Aging is the major risk factor for developing metabolic dysfunction and involves a massive reprograming of cell-type-selective epigenomes linked to altered gene expression and metabolic pathways. Intriguingly, alterations of HDAC3 chromatin occupancy and co-repressor complex function may play a particular role in the age-dependent epigenome reprograming of hepatocytes linked to metabolic dysfunction, in particular to steatohepatitis and hepatic inflammation (79). As underlying mechanism the study suggests that in the livers of young mice (3 months) the HDAC3 co-repressor complex at PPAR and LXR targets was sufficient to regulate lipid metabolism according to the circadian rhythm. However, in the livers of older mice (21 months) loss of HDAC3 chromatin occupancy caused increased histone acetylation and NR activation. Specifically, the loss of HDAC3 triggered the gain of FOXA2 at the regulatory elements, which further cooperated with PPARα to upregulate genes involved in lipid synthesis and storage. The study suggests that this reciprocal binding of FOXA2 and HDAC3 contributes to the de-repression of PPAR and LXR and thereby triggers aging-related hepatosteatosis (79). While the study has uncovered a potentially intriguing role of HDAC3 and the co-repressor complex in reprogramming the liver epigenome during aging in mice, there are many open issues and implications that deserve further investigation. Although the age-dependent dysfunction of HDAC3 may be in part linked to its association with the nuclear lamina, additional factors may contribute to the loss of chromatin occupancy during aging. For example, does HDAC3 expression in hepatocytes change during aging, and if so what are the underlying mechanisms? Do PTMs change HDAC3 function including its association with chromatin and the lamina, and if so how are these PTMs regulated? Finally, are the implications derived from mouse studies also relevant to humans, in particular to explain the link between aging, co-repressor complex dysfunction, hepatocyte epigenome alterations, and NAFLD/NASH?

### The Liver HDAC3 Complex Associates With the Co-repressor PROX1

HDAC3 interactome analysis by mass spectrometry identified the previously known NR co-repressor and homeodomain TF PROX1 as a co-repressor complex- associated factor (41). The study demonstrated that in hepatocytes HDAC3 and PROX1 extensively co-occupy regulatory promoters and enhancers of metabolic genes, and that their chromatin binding was remarkably reduced upon depletion of HNF4α. Consistently, liver depletion of PROX1 and HDAC3 increased hepatic TG. These data suggest a model thereby the co-repressor complex is recruited to chromatin by HNF4α via the HDAC3-PROX1 module (Figure 4E). This recruitment seems to occur independently of RevErb since the REV-DR2 motif was only enriched in HDAC3-selective peaks while not present in shared HDAC3-PROX-1 peaks (41). Overall, the study identified a probably hepatocyte-specific co-repressor sub-complex in which a HDAC3-PROX-1 module selectively docks to HNF4α as the major target TF in hepatocytes.

### Evidence That the Enzymatic Deacetylase Function Is Dispensable for HDAC3 Function

As discussed above, HDAC3 genome binding was significantly reduced and its deacetylase enzyme activity was barely detectable in NCOR/SMRT DADm mice. However, these mice did not simply phenocopy the effects of HDAC3 depletion on hepatic lipid metabolism, neither at the transcriptional not at the physiological level. HDAC3 LKO mice showed more dramatic effects on lipogenesis, more severe hepatosteatosis as well as disrupted cholesterol homeostasis (34, 38). In contrast, DADm mice exhibited fewer and milder changes in lipogenesis, moderate hepatic steatosis, and no alteration in hepatic cholesterol (34). In addition, while global DADm knockin mice live to adulthood, global genetic KO of NCOR, SMRT or HDAC3 all caused embryonic lethality (17, 30, 35). Together, this implies that the DAD, and thus the enzymatic activity of HDAC3, may not be essential for fulfilling key repressive functions of the co-repressor complex, such as those linked for embryonic development (26).

To further characterize the non-enzymatic role of HDAC3, several deacetylase-dead HDAC3 mutants were introduced into HDAC3-LKO mouse livers using hepatocyte-specific AAV vectors, To test whether the HDAC3 LKO phenotype could be reversed by this approach, two distinct types of mutations were used to eliminate deacetylase activity: First, mutations of catalytically essential residue(s) without affecting the association with DAD (such as Y298F, H134A/H135A) (26), and second, mutations of the key residues required for the interaction with DAD which further disrupt the deacetylase activity (such as K25A) (26, 34) (Figure 2). Remarkably, all mutants were able to restore the repression of most lipogenic genes, which were upregulated in HDAC3 LKO livers. Consistently, all HDAC3 mutants also reversed the hepatosteatosis phenotype. Surprisingly, WT and mutant HDAC3 displayed a similar chromatin occupancy, suggesting that the DAD domain was dispensable for HDAC3 interactions and pointing at an alternative interaction domain. Indeed, this was demonstrated by a novel, completely non-functional HEBI mutant of HDAC3. Overall, the main conclusion of the study was that the *in vivo* repressive function of HDAC3 in liver, albeit independent of deacetylase activity, fully depends on the interaction with NCOR, while SMRT seems dispensable for liver HDAC3. HDAC3 thus functions not independently but largely as a subunit of the NCOR-containing co-repressor complex (26).

## TBL1 and TBLR1 Regulate Repression and Activation Pathways in Liver

### TBL1 and TBLR1 Function as Unique Co-repressor-Co-activator Exchange Factors

Structure data suggest that TBL1 and its homolog TBLR1 directly interact with one of the RD domains of NCOR/SMRT, and simultaneously with GPS2 via their N-terminal region (12, 13). TBL1/TBLR1 appear to bind hypoacetylated histones H2B and H4, which could be essential to keep the co-repressor complex stably chromatin-associated, in addition to its recruitment by TFs, and to adequately repress transcription (9, 12, 80) (Figure 2). In earlier studies using Hela cells, simultaneous knock-down of TBL1 and TBLR1 abolished TR repression, supporting their essential role as co-repressor subunits in mediating repression by unliganded TR (9, 12). However, in the presence of ligand, knock-down of TBL1 and TBLR1 also completely abolished TR activation, suggesting them to alternatively function as co-activators (12, 81). Indeed, upon ligand treatment, TBL1/TBLR1 remained chromatin-bound along with co-activators, while NCOR and SMRT were dismissed. Mechanistically, is has been suggested that TBL1/TBLR1 serve as NR co-repressor-co-activator-exchange factors, which in the presence of ligands recruit the ubiquitin conjugating/19S proteasome complex to trigger ubiquitination-dependent dismissal of the co-repressor complex and subsequent co-activator recruitment to chromatin (81, 82).

### TBL1 Controls Steatohepatitis in Part via PPARα Activation Pathways

This dual function of TBL1 and TBLR1 has to be taken into account then trying to identify mechanisms underlying the respective KO or RNAi-knockdown mouse models in liver (42). One study has discovered that in the livers of obese/diabetic mice (*db/db* mice and HFD-fed mice) expression of TBL1, but not of TBLR1, was impaired by fatty acids as evidenced by the reduced level of TBL1 mRNA and protein. Notably, reduced TBL1 mRNA expression significantly correlated with increased liver TG content in a human NAFLD cohort (42). To identify the physiological role of TBL1 and TBLR1 in liver, TBL1/TBLR1-deficient mouse models were established using adenovirus, to express shRNA, or AAV, to express miRNA, under control of a hepatocyte-specific promoter. Loss of hepatic TBL1 resulted in increased hepatic TG accumulation, serum very low-density lipoprotein (VLDL) and TG levels (in the fed/fasted states under chow diet and HFD), and in decreased levels of ketone bodies in the serum, demonstrating that hepatic TBL1 deficiency triggers lipogenesis while blunting fatty acid oxidation (42) (Figure 3B). Surprisingly, these mice display mildly improved systemic glucose tolerance and insulin sensitivity without changes in body weight even with hepatosteatosis, similar to HDAC3 LKO phenotype (37, 42).

Despite the differences in the liver regulation of their mRNA expression, TBL1 and TBLR1 seem to have synergistic functions in regulating hepatic lipid metabolism since hepatic TBLR1 loss phenocopies TBL1 deficiency. Simultaneously ablation of both TBL1 and TBLR1 triggered much more severe hepatosteatosis and much lower ketone bodies release than single knock-down. Intriguingly, these effects were gone in PPARα KO mice, demonstrating TBL1 and TBLR1 synergistically prevent hepatic steatosis and hypertriglyceridemia by regulating fatty acid oxidation genes in a PPARα-dependent manner (42). In line with the co-regulator exchange function, TBL1/TBLR1 deficiency triggered the release of known PPARα co-activators and promoted the recruitment of NCOR and HDAC3 to the promoters of fatty acid oxidation genes (42). The liver studies highlight that TBL1 and TBLR1, unlike the other core subunits of the corepressor complex, function also as context-dependent co-activators in hepatocyte pathways.

### TBLR1 Controls Steatohepatitis in Part via LXR Pathways

The peculiar co-activator function was independently demonstrated for TBLR1 in two independent studies analyzing liver LXR pathways. The first study analyzed the requirement of the co-repressor core subunits for LXR activation in human hepatocytes and found that depletion of TBLR1 but not of TBL1 reduced ligand-dependent LXR activation of key target genes (83). Interestingly, TBLR1 seems to cooperate with GPS2 (discussed further below) in this LXR pathway, although at mechanistically distinct steps. The second study analyzed knockin mice carrying a LXRα S196A phosphorylation-defective mutant (84). Upon a high-fat/high-cholesterol diet, mutant mice exhibited enhanced hepatic steatosis but impaired hepatic inflammation and fibrosis. They were protected from dietary cholesterol accumulation by reprogramming hepatic inflammatory and metabolic gene expression. Analysis of the LXRα S196A liver transcriptome revealed an increased expression of lipogenic genes and a robust repression of pro-inflammatory and pro-fibrotic genes. Investigation of the mechanisms accounting for the differential expression of these diet-sensitive genes revealed that liver TBLR1, along with NCOR, had a higher binding affinity to the LXRα S196A mutant than to the WT LXRα, as judged by mass spectrometry. Consistently, TBLR1 recruitment to LXR-binding sites at chromatin was enhanced in LXRα-S196A livers, suggesting a key role of TBLR1 in sensing the diet-induced LXR phosphorylation switch and transforming it into altered gene expression (84). Overall, these studies emphasize the unique role of the co-repressor complex core subunits TBL1 and TBLR1 in activation pathways governed by NRs.

## GPS2-NR Interactions Specify Repression of Metabolic and Inflammatory Pathways in Hepatocytes

### Identification of GPS2 as PPARα-Associated Protein From Liver

GPS2 was initially cloned as a human cDNA encoding a potential human suppressor of conserved G-protein pathways in yeast (85), suggesting functions in intracellular signaling. GPS2 was independently identified as a NR-associated protein, along with the co-repressors NCOR, SMRT, and RIP140 (NRIP1) in yeast two-hybrid screenings from liver cDNA libraries using PPARα as bait (16), and using the orphan receptor SHP (86). GPS2 was lateron biochemically purified as a NCOR/SMRT/HDAC3 co-repressor complex subunit and suggested to be involved in both NR repression and anti-inflammatory crosstalk (11).

### GPS2 Protein Structure, Modifications, and Mutations

GPS2 is a ubiquitously expressed 37 kDa protein, containing 327 aa. The N-terminal coiled-coil domain (aa 1–90) is sufficient to simultaneously interact with NCOR/SMRT as well as with TBL1/TBLR1, thereby forming a three-way co-repressor complex core structure (13). Importantly, GPS2 interacts with several liver NRs (e.g., PPARα, LXRs, SHP), and inflammatory TFs (e.g., c-Jun) by its C-terminal domain (aa 100–327) (44, 83, 86, 87), thus serving as a TF-binding subunit of the co-repressor complex, in addition to NCOR and SMRT (Figure 2). While metabolic signals that reversibly control GPS2 expression in the liver have not yet been identified, PTMs seem to play critical roles in regulating the protein function of GPS2. Up to now, methylation (R312, R323) (88–90), ubiquitylation (K66) (91), and SUMOylation (K45, K71) (92) of GPS2 have been reported. Further, GPS2 mutations located in the N-terminal coiled-coil domain were found in the context of human cancers such as medulloblastoma, supporting the role of this domain for appropriate co-repressor complex function (93). Whether GPS2 mutations play also a role in human metabolic diseases such as NAFLD is currently not known.

### NR-Independent Anti-inflammatory GPS2 Actions in Macrophages and Adipocytes

Both *in vivo* and *in vitro* studies indicate that GPS2 has multiple functions in various aspects of metabolic and inflammatory regulation (8). Many of these functions are consistent with a role of GPS2 as a core subunit of the NR co-repressor complex, while others point at independent roles in transcriptional activation, gene-and cell-type-selective, and even in non-genomic signaling. For example, cell line-based studies suggest that GPS2 participates in macrophage cholesterol efflux via positively regulating the expression of genes encoding the two key cholesterol transporters *ABCA1* and *ABCG1*, albeit by utilizing two distinct mechanisms and targets (83, 94). In human macrophages, GPS2 is selectively required to facilitate LXR-dependent *ABCG1* expression, indicative of a “co-activator” role (83). In mouse and human macrophages, GPS2 cooperates with the lipopolysaccharide (LPS)-inducible Nuclear Factor kappa B (NF-kB) subunit p65 to activate *ABCA1* gene expression, while it seemed not involved in LXR activation of this gene. The intriguing role of GPS2 in mediating transcriptional crosstalk between cholesterol efflux and inflammation was demonstrated to involve cooperation of GPS2 with the other core subunits of the co-repressor complex (94). The key anti-inflammatory role of GPS2 as epigenome modifier preferentially targeting inflammatory AP-1 pathways was discovered using macrophage-specific GPS2 KO mice along with genomic investigations in tissue macrophages and in the mouse macrophage RAW cell line. The study identified a GPS2-SMRT containing sub-complex as an epigenomic component of metabolic adipose tissue macrophage activation in the context of obesity and T2D (14). In sum, these macrophage GPS2-focused studies have conceptually advanced our understanding of the individual roles of each subunit of, what we assume to be, one co-repressor complex (8).

In human adipose tissue, both in adipocytes and infiltrating macrophages, expression of GPS2 was found to be down-regulated in obese subjects, and GPS2 expression was inversely correlated to the diabetic status and the expression of key inflammatory genes (14, 95). The anti-inflammatory role of GPS2 in adipocytes was further confirmed *in vivo* in aP2-GPS2 transgenic mice, as evidenced by the significantly reduced JNK1/2 activation and abrogated expression of inflammatory genes (*Il-12*β and *Ccl2*) (96). A cytoplasmic role of GPS2 was suggested to be required for JNK suppression by inhibiting the TNF-receptor associated factor 2 (TRAF2)/Ubc13 enzymatic activity upon stimulation with tumor necrosis factor alpha (TNFα) (96). Recent studies using adipocyte-specific GPS2 KO mice revealed additional pathways to be affected, such as HIF1α pathways and mitochondrial biogenesis, but the underlying genomic vs. non-genomic mechanisms remain to be clarified (97–99). So far it is probably safe to state that both in macrophages and adipocytes GPS2 seems largely, but not exclusively, to cooperate with SMRT to function within an anti-inflammatory co-repressor complex targeting inflammatory and other TFs, but surprisingly few NRs (e.g., PPARγ-regulated lipolysis in adipocytes).

### NR-Dependent Metabolic and Anti-inflammatory GPS2 Actions in Hepatocytes

Several studies have begun to analyze the role of GPS2 in mouse and human hepatocytes. They revealed that GPS2, via interacting with different NRs (such as PPARα, LXR, FXR, LRH1, HNF4α, and SHP), plays important roles in metabolic and inflammatory regulation of liver pathways, some of which are involved in NAFLD/NASH (44, 86, 87). The two initial studies revealed that GPS2 serves as a physiological co-regulator of cholesterol homeostasis by affecting cholesterol to bile acid biosynthesis in the liver (86) and by participating in cholesterol transport and efflux in hepatocytes and macrophages via ABCG1 (83). Activation of FXR initiates a feedback regulatory loop via induction of the atypical orphan receptor and co-repressor SHP (discussed further below), which suppresses LRH1- and HNF4α-dependent expression of *CYP7A1* and *CYP8B1*, the two major enzymes for bile acid synthesis. The first study suggested that GPS2 regulates these genes by two separate mechanisms in opposite ways. At the *CYP7A1* promoter, GPS2 serves as the bridging subunit to connect the co-repressor complex with SHP, thereby triggering repression. However, at the *CYP8B1* enhancer and promoter, GPS2 seems required for the recruitment of co-activators, thereby triggering activation (86) (Figure 4F). The second study found that GPS2 is selectively required to facilitate LXR-induced *ABCG1* expression in human hepatocytes, while having no effect on LXR-induced *ABCA1* expression (83). This highlights the fundamental molecular differences between transcriptional regulatory elements of two related key LXR target genes encoding cholesterol transporters. By dismissing G9a and recruiting histone de-methylases (KDMs) and acetyltransferases (HATs) to trigger H3K9 demethylation and subsequent H3 acetylation, GPS2 may prime an appropriate local chromatin environment to facilitate ligand-induced LXR recruitment and promoter-enhancer communication (83). Since GPS2 in that mechanism promotes the chromatin access of a target TF, GPS2 may exert an unusual “pioneer-type” function, distinct from classic co-activators. Indeed, a highly related GPS2 mechanism has been subsequently identified for PPARγ in adipocytes (100).

The anti-inflammatory role of GPS2 can be exemplified by GPS2-dependent actions of LRH1 and LXRβ in trans-repression of the hepatic acute phase response (87). Importantly, GPS2 was identified as a sensor of SUMOylated LRH1 and LXRβ in hepatocytes. This provided a missing link in the trans-repression model, explaining how the NCOR-containing co-repressor complex can be recruited to ligand-activated NRs. Specifically, the study has demonstrated that the GPS2-SUMO-NR complex maintains repression even upon IL-1β and IL-6 stimulation, thereby inhibiting inflammatory gene expression during inflammation and infection (Figure 3G). The study also revealed that in SUMO-1 KO mice the acute phase response was increased, which may be caused by diminished LRH1 SUMOylation (87).

### GPS2 Triggers Steatohepatitis by Repressing PPARα in Mice, and Perhaps in Humans

Our recent work utilizing liver-specific GPS2 KO mice uncovered a hitherto unknown role of GPS2 as an epigenetic modulator in hepatocytes that represses PPARα-dependent lipid catabolism and thereby promotes the development of NAFLD (44). The development of a conditional LKO model was necessary since the global GPS2 KO caused embryonic lethality (43), similar to global KO of NCOR (17), SMRT (30), and HDAC3 (35), indicating that GPS2 along with its associated complex subunits plays critical roles in development. In the conditional LKO model we found that loss of GPS2 caused the activation of fatty acid oxidation genes, known to be controlled by PPARα (Figures 3B, 4H). Loss of PPARα repression in LKO mice improved liver steatosis upon HFD feeding and improved fibrosis upon feeding a methionine-choline-deficient diet (MCD), due to increased lipid burning as detected by elevated ketone body levels. The study also provides a unique resource of hepatocyte ChIP-seq data as we determined chromatin occupancy for GPS2, NCOR, and PPARα, along with the H3K27ac enhancer mark, in livers of WT mice and in the respective KO livers depleting each of these factors. Amongst the intriguing findings were (i) that loss of PPARα caused release of GPS2 and NCOR from chromatin, (ii) that loss of NCOR caused release of GPS2 from chromatin, but not vice versa, and (iii) that loss of GPS2 led to a destabilization of PPARα-NCOR interactions, although not being sufficient for causing NCOR release from chromatin.

Remarkably, the protective phenotype of the GPS2 LKO mice is unique amongst hitherto described liver co-regulator KO mouse models in the context of NAFLD as it is the only model which improved diet-induced fatty liver disease instead of worsening it. Further, this hepatic function of GPS2 appears to be conserved between mice and humans as GPS2 mRNA levels correlated with fibrogenic and inflammatory gene expression in human NAFLD/NASH livers. This study might thus provide hepatocyte-based epigenetic explanations for the diverse susceptibility in NAFLD/NASH patients to develop more severe stages of liver fibrosis and ultimately liver cancer, in addition to alterations in other cell types such as liver-resident immune cells (2).

## Perspectives

In this review, we have summarized our current understanding of the multiple functions of a fundamental co-repressor complex in modulating liver metabolism and disease. The particular emphasis on pathways controlled by this co-repressor complex reflects the origin of discovery, the fact that many liver NRs are the main targets, and the current focus of the field as evidenced from the published studies. Many of these studies have generated and characterized liver-specific loss-of-function mouse models, which revealed phenotypes linked to pathways controlled by the key metabolic NRs. Some of these studies have additionally integrated state-of-the-art next generation sequencing approaches such as ChIP-seq. These approaches are still challenging for co-regulators, which do not bind DNA directly and for most of them high-quality antibodies are not commercially available. Future research has to overcome this problem and generate reproducible data to compare and validate different studies. Also, very few co-repressor-focused studies have so far integrated human data and compared mouse and human liver pathways both at the physiological and (epi)genomic levels. Specifically, further studies are needed to better understand the functional cooperation or diversification of the individual co-repressor complex subunits in liver pathways, including those conserved between mouse and human hepatocytes. Despite the high evolutionary conservation of co-repressors and their complexes, their genomic targets, i.e., the regulatory promoters and enhancers, can be highly divergent between humans and mice. Furthermore, we should study much more the signal-regulated PTMs that potentially play a major role in the target selection and the co-repressor/co-activator switch. These issues are highly relevant for the better understanding and future targeting of liver disease pathways triggered by aging, nutrition and life style, such as obesity-associated NAFLD and hepatic insulin resistance. Pathway-specific therapeutic intervention using rapidly evolving RNA/protein-targeting technologies may be possible in near future, but research efforts utilizing humanized liver disease models should before scrutinize the pros and cons of targeting hepatic TF-co-regulator networks.

## Author Contributions

NL and ET collected information and wrote the manuscript. All co-authors contributed to text editions, database search, and design of Figures and Table. TJ prepared the Figures with the help of NL.

### Conflict of Interest Statement

The authors declare that the research was conducted in the absence of any commercial or financial relationships that could be construed as a potential conflict of interest.

## Abbreviations

AAV: Adeno-associated virus
ABCA1: ATP binding cassette subfamily A member 1
ABCG1: ATP-binding cassette subfamily G member 1
AICAR: 5-Aminoimidazole-4-carboxamide ribonucleotide
Alb: Albumin
DAD: Deacetylase-activating domain
ERRα, Estrogen-related receptor alpha, nuclear receptor subfamily 3, group B: member 1
FOXA2, Forkhead box protein A2: hepatocyte nuclear factor 3-beta
FXR, Farnesoid X receptor, nuclear receptor subfamily 1, group H: member 4
GPS2: G-protein pathway suppressor 2
HDAC: Histone deacetylase
HFD: High-fat diet
HID: Histone-interacting domain
HNF4α, Hepatocyte nuclear factor 4 alpha, nuclear receptor subfamily 2, group A: member 1
HNF6: Hepatocyte nuclear factor 6
RID: Nuclear receptor-interacting domain
JNK: c-Jun N-terminal kinase
KO: Knock-out
LDL: Low-density lipoprotein
LKO: Liver/hepatocyte-specific knockout
LRH1, Liver receptor homolog 1, nuclear receptor subfamily 5, group A: member 2
LXRα, Liver X receptor alpha: nuclear receptor subfamily 1 group H member 3
LXRβ, Liver X receptor beta: nuclear receptor subfamily 1 group H member 2
NAFLD: Non-alcoholic fatty liver disease
NASH: Non-alcoholic steatohepatitis
NCOR, Nuclear receptor co-repressor, alias N-CoR: NCOR1 NR
PPARα, Nuclear receptor Peroxisome proliferator-activated receptor alpha, nuclear receptor subfamily 1, group C: member 1
PPARδ, Peroxisome proliferator-activated receptor delta, nuclear receptor subfamily 1, group C: member 2
PPARγ, Peroxisome proliferator-activated receptor gamma, nuclear receptor subfamily 1, group C: member 3
PROX1: Prospero-related homeobox protein 1
PTM: Post-translational modification
PXR, Pregnane X receptor, nuclear receptor subfamily 1, group I: member 2
RAR: Retinoic acid receptors, nuclear receptor subfamily 1, group B, members 1,2,3
RD: Repression domain
RevErbα: Nuclear receptor subfamily 1, group D, member 1
RevErbβ: Nuclear receptor subfamily 1, group D, member 2
RID: Receptor (NR)-interacting domain
RIP140: Receptor-interacting protein 140, alias NRIP1 (nuclear receptor-interacting protein 1)
RXR: Retinoid X receptors, nuclear receptor subfamily 2, group B, members 1,2,3
SANT: SW13/ADA2/NCoR/TFIIB
SHP: Small heterodimer partner, nuclear receptor subfamily 0, group B, member 2
SMRT: Silencing mediator of retinoic acid and thyroid hormone receptor, alias NCOR2
SREBP: Sterol regulatory element-binding protein
SUMO: Small ubiquitin-like modifier
T2D: Type 2 diabetes
TBL1: Transducin β-like protein 1
TBLR1: Transducin β-like protein-related 1
TF: Transcription factor
TG: Triglycerides
TH: Thyroid hormone
TR: Thyroid hormone receptors, nuclear receptor subfamily 1, group A, members 1, 2
VLDL: Very low-density lipoprotein
WT: Wild-type.
